# Improving High Risk Care Management Education in the Outpatient Setting

**DOI:** 10.1093/geroni/igaf122.3268

**Published:** 2025-12-31

**Authors:** Fiorella Perez, Stephanie Chow, Danialle Coyne, Jodi Payne, Lizette Andrango, Susana Lavayen

**Affiliations:** Icahn School of Medicine at Mount Sinai, New York, New York, United States; Icahn School of Medicine at Mount Sinai, New York, New York, United States; Mount Sinai Hospital, New York, New York, United States; Icahn School of Medicine at Mount Sinai, New York, New York, United States; Mount Sinai Hospital, New York, New York, United States; Mount Sinai Hospital, New York, New York, United States

## Abstract

The Aging, Life Innovations, Goals & Needs(ALIGN) Program is an innovative intensive ambulatory model of care that manages older “high risk” patients at risk for acute health decline with a thoughtful curriculum designed to highlight approaches to geriatric medical and psychosocial care. ALIGN curriculum (AC) objectives include learning skills in identifying high risk patients and developing a comprehensive geriatric care plan using the 5Ms of Geriatrics framework, plus “Maintaining Care.” Fellows were assigned a small patient panel and participated in team huddles. At rotation end, fellows provided a written sign-out of patients. Following the 2-week AC, fellows completed a post-rotation survey. Six fellows completed the survey between June 2021 and June 2022, rating usefulness (Scale 0-5) of online education, team huddles, shadowing team and confidence in managing high risk patients. Results showed fellows appreciated active 1:1 time with attendings. Subsequently, 13 post-rotation surveys were collected from June 2022-January 2024. Shadowing the team was rated most useful (53.8%) as was seeing patients independently (69.2%). Fellows rated their confidence (scale 0-5) in screening for high-risk older patients as high (average 4.3). Moreover, 61.5 % fellows found 1:1 ALIGN attending education useful, as well as 1:1 educational time with the social worker and care coordinator. For many, ALIGN was their first experience in high-risk patient care management. Likelihood to recommend the ALIGN rotation was 6.9 out of 10. In conclusion, AC is beneficial in promoting leadership and innovation for trainees amidst a perpetually changing landscape in geriatric care.

